# Non-allergenic factors from pollen modulate T helper cell instructing notch ligands on dendritic cells

**DOI:** 10.1186/s40413-014-0054-8

**Published:** 2015-01-20

**Authors:** Stefanie Gilles, Isabelle Beck, Stefan Lange, Johannes Ring, Heidrun Behrendt, Claudia Traidl-Hoffmann

**Affiliations:** Institute of Environmental Medicine, UNIKA-T, medical faculty of the Technische Universität München, Augsburg, Germany; CK-Care, Christine Kühne Center for Allergy Research and Education, Davos-Wolfgang, Switzerland; Department of Dermatology and Allergy Biederstein, Technische Universität München, Munich, Germany; ZAUM – Center for Allergy and Environment, Helmholtz Center and Technische Universität München, Munich, Germany

**Keywords:** Dendritic cells, MyD88, Notch ligands, Pollen

## Abstract

**Background:**

Pollen allergens are delivered to epithelial surfaces of the upper respiratory tract in conjunction with multiple endogenous adjuvants. We previously demonstrated pollen-mediated modulation of cytokine and chemokine production of dendritic cells, contributing to a Th2-dominated micromilieu. As T helper cell differentiation not only depends on dendritic cell-derived cytokines but also on cell-cell-contact mediated mechanisms, we studied the expression of notch ligands and myeloid differentiation primary response protein 88 (MyD88) in dendritic cells matured in the presence of aqueous birch pollen extracts and pollen-associated E_1_-phytoprostanes.

**Methods:**

Human monocyte-derived dendritic cells were stimulated with aqueous birch pollen extracts in the absence or presence of lipopolysaccharide, and mRNA expression levels of notch ligands *delta-1* and −*4*, jagged-1 and −*2* and of *myd88* were determined. Regulation of Delta*-4* and MyD88 by aqueous pollen extracts was assessed on protein level. The contribution of notch signaling to T helper cell differentiation was analyzed in allogeneic T cell stimulation assays.

**Results:**

In immature dendritic cells, stimulation with pollen extracts resulted in an induction of both *delta* and *jagged* notch ligands. The lipopolysaccharide-induced up-regulation of *delta-1* and −*4* and of *myd88* was decreased by aqueous pollen extracts, whereas *jagged* expression was induced. Reduction of Delta-4 and MyD88 by aqueous pollen extracts was confirmed on protein level. The Th2-skewing activity was contained in a fraction of aqueous pollen extracts enriched for molecules <3 kDa and was distinct from the previously identified E_1_-phytoprostanes. Reduction of notch signaling in dendritic cells matured in the presence aqueous pollen extract leads to inhibition of IL-10 and to induction of IL-5 production in naïve T cells differentiated by these dendritic cells.

**Conclusions:**

Pollen derived, non-allergenic factors reduce the dendritic cell’s expression of Th1 instructing Delta-like notch ligands and of MyD88, thereby promoting Th2 skewing of T helper cell responses.

**Electronic supplementary material:**

The online version of this article (doi:10.1186/s40413-014-0054-8) contains supplementary material, which is available to authorized users.

## Introduction

The development of allergic sensitization is attributed to specific proteins – the allergens – derived from the allergen carrier, e.g. pollen grains. Dendritic cells play a pivotal role in allergic sensitization [1,2]. As sentinels in the tissues, they constantly take up and process host and foreign antigens. Under inflammatory conditions, they are activated and undergo a fundamental reprogramming enabling them to migrate to the regional lymph nodes, where they instruct naïve CD4^+^ T cells to differentiate into effector cells. It is a generally accepted concept that the dendritic cell transmits a complex integrated signal to the T cell induced by pathogen, adjuvant factors and tissue environment. This, in turn, determines which type of T helper cell response is mounted [3]. In the case of pollen, the natural consequence of exposure is tolerance. However, in susceptible individuals a Th2-dominated, allergen-specific immune response evolves – the underlying reasons being inexplicit to date. Previously, non-allergenic adjuvant factors derived from pollen grains were shown to contribute to the allergenicity of pollen. Those adjuvant factors can act both in a proinflammatory and in an immune-modulatory way on cells involved in the allergic cascade (reviewed in [4,5]). The concept of adjuvant factors follows the evident fact that pollen do not only release the allergen. Rather, pollen allergens are delivered to epithelia in conjunction with multiple other protein and non-protein factors such as bacterial and fungal cell-wall components, enzymes, and the previously identified pollen-associated lipid mediators (PALMs) [3,5-9]. Taken together, these factors seem to signal danger to the tissue, ultimately leading to sensitization and Th2-dominated allergic immune responses in susceptible individuals. Up to now this adjuvant activity was attributed to pollen-derived linolenic and linoleic acid derivatives. A subgroup of PALMs, the E_1_-phytoprostanes (PPE_1_), were shown to block the DC’s capacity to secrete proinflammatory and Th1 cytokines and to prime Th1 responses *in vitro* and *in vivo* [10-13].

Besides DC cytokines like IL12 [14], the outcome of the T helper cell differentiation process depends on the interaction of notch isoforms on the T cell and distinct notch ligands on the dendritic cell [15]. In fact, notch signaling in T cells is critically required for Th1 differentiation [16]. On the antigen-presenting cell, notch ligands *delta like-1* and −*4* have been shown to deliver Th1 instructing signals [15], which act independently of IL12 secretion. In contrast, loss of *delta like-4* and up-regulation of *jagged-1* and −*2* promote Th2 differentiation [15,17]. Interaction of notch on the T cell with its cognate ligand on the DC leads to cleavage of notch intracellular domain, which translocates to the nucleus and interacts with the transcriptional repressor/activator RBPJκ. In cooperation with coactivators of the Mastermind-like (MAML) family, this complex activates the genes of the key transcription factors Tbet or GATA3, which then drive Th1- or Th2-differentiation, respectively [18].

In murine bone marrow-derived DCs, the upregulation of *delta like* notch ligands upon Toll-like receptor (TLR)-engagement is crucially dependent on the expression of functional MyD88 [15]. In the absence of the signaling adapter MyD88, TLR engagement induces *jagged* but not *delta like-4*, resulting in Th2-promoting signals. Some micromilieu factors, e. g. PGE_2_, also induce upregulation of *jagged* by pathways still to be elucidated (reviewed in [18]). We were thus interested in investigating whether non-allergenic pollen-derived factors modulate the expression of MyD88 and notch ligands in dendritic cells and if so, whether the previously characterized E_1_-phytoprostanes might be responsible for this modulation.

## Methods

### Subjects

Healthy, non-atopic volunteers (aged 20–41 years) were screened for total serum IgE levels and for specific IgE against common allergens as described before [10]. Non-atopic blood donors were characterized by low total serum IgE (<20 kU/L) and a negative history for allergic diseases. The ethical commitee of the Technische Universität Munich approved the study and volunteers were enrolled after written informed consent.

### Aqueous pollen extracts (APE)

Aqueous birch pollen extracts were prepared as described before [19]. Commercial pollen (Allergon) and self-collected pollen specimens were used for the preparation of the extracts. To obtain allergen-free APE fractions, the total extracts were ultra-filtrated using 3 kDa cutoff filters (Amicon ultra YM3, Millipore, Schwalbach, Germany). Content of Bet v 1, the major allergen of birch pollen was below detection level as determined by ELISA (Additional file 1: Figure S1). The concentrations of APE given in text and figures correspond to the amount of pollen used to generate the extract in a given volume (e. g. 10 mg/mL = extract of 10 mg pollen per mL DC medium).

### Reagents

Ultra-pure *E. coli* LPS was purchased from Invivogen, Toulouse, France, PGE_2_ from Cayman Chemicals, Ann Arbor, MI, USA. PPE_1_ was supplied as a 1:1 mixture of two regio-isomeres prepared by autoxidation of α-linolenic acid and purified as described before [13].

### Culture of monocyte-derived dendritic cells

PBMCs were isolated from peripheral blood by densitiy gradient centrifugation. CD14^+^ monocytes were purified by MACS (Miltenyi Biotech, Bergisch Gladbach, Germany) and cultured in DC medium (RPMI-1640, 10% FCS, 2 mmol/L L-glutamine, 20 μg/mL gentamycin, 500 μmol/L 2-mecaptoehanol) in the presence of 50U/mL rhGM-CSF and 50U/mL rhIL-4 (PormoCell, Heidelberg, Germany). Immature monocyte-derived DCs harvested on day 5 were >95% pure as assessed by flow cytometry (CD14^−^ CD1a^+^ HLA-DR^+^ CD80^low^ CD83^−^ CD86^low^ CD40^low^). Antibodies for flow cytometry were from BD Pharmingen, Heidelberg, Germany and eBioscience, Heidelberg, Germany.

### Quantitative mRNA analysis

Total RNA was extracted from DCs after 12 hours incubation with the indicated stimulants by phenol/chloroform extraction using peqGOLD RNAPure buffer (Peqlab, Erlangen, Germany). RNA was reverse transcribed using High-Capacity cDNA Reverse Transcription Kit (Applied Biosystems, Darmstadt, Germany) following the manufacturers instructions. The cycler protocol was: 1×(10 min 25°C); 1×(120 min 37°C); 1×(5 sec 85°C); 1×(∞ 4°C). PCR-reactions for dll1, dll4, jagged2 and myd88 (Assay on demand, Applied Biosystems, Darmstadt, Germany) were run on an ABI PRISM® 7000 Sequence Detection System device (Applied Biosystems Division of Perkin Elmer, Foster City, CA, USA) using the following program: 1×(2 min 50°C); 1×(10 min 95°C); 45× [(15 sec 95°C); (1 min 60°C)]. 18S RNA, GAPDH and EF1α served as house-keeping genes. Relative expression was calculated by the 2^-ΔΔCT^ method [20].

### Analysis of surface protein expression on denritic cells by flow cytometry

Immature monocyte-derived dendritic cells (DCs) were incubated in the presence of medium, *Staphylococcus aureus* lipopolysaccharide (LPS; 100 ng/mL) or LPS plus aqueous birch pollen extract (APE, 10 mg/mL). After 24 h, cells were washed and stained with PE-conjugated mouse anti-human Dll4 (Biolegend, San Diego, CA, USA) or matched isotype control antibody. Maturation markers were stained with antibodies against Dll4 (PE), CD1a (APC), CD80 (FITC), CD83 (PE), CD86 (APC), CD40 (FITC), CCR-7 (PE), HLA-DR (APC), B7-DC (PE) and B7-H1 (APC), all from BD Pharmingen, Heidelberg, Germany. Mean fluorescence intensities (MFIs) of matched isotype controls were subtracted, respectively. Cells were acquired on a FACScalibur (Becton Dickinson, Heidelberg, Germany).

### Detection of MyD88 protein by Western Blotting

Monocyte-derived dendritic cells were incubated for 24 h in the presence of medium, LPS (100 ng/mL), LPS plus aqueous pollen extract (APE, 10 mg/mL) or LPS plus APE < 3 kDa (10 mg/mL). Cytoplasmic extracts were prepared using the Nuclear Extract Kit (Active Motif, Carlsbad, CA, USA), following the manufacturers instructions. 20 μg total protein were subjected to SDS-PAGE under reducing conditions and transferred to a nitrocellulose membrane by Western Blotting. For detection of MyD88, antibodies used were Purfied Anti-Human MyD88 (eBioscience, Heidelberg, Germany; dilution 1:500) and goat anti-rabbit IgG-HRP (Santa Cruz Biotechnology, Santa Cruz, CA, USA; dilution 1:5000). β-actin was detected by mouse anti-human β-Actin and donkey anti-mouse IgG-HRP (both from Santa Cruz; dilution 1:5000). Intensities of bands were calculated using ChemoStar™ software (Intas, Goettingen, Germany).

### Allogeneic T cell stimulation assays

Immature monocyte-derived DCs were harvested at day 5 of culture and stimulated with different concentrations of aqueous birch pollen extracts (APE; 1 mg/ml, 3 mg/ml, 10 mg/ml) or a Th2-differentiating cocktail: LPS (100 ng/ml), PGE_2_ (10 μM), neutralizing anti-IL-12 (25 ng/ml) and anti-IFN-γ (1 μg/ml) antibodies. After 24 h, stimulated DCs were washed twice with PBS and seeded into 96-well plates at a density of 10,000 cells per well. Allogeneic, naïve CD4^+^ T cells were added to the DCs at a density of 100,000 cells per well (DC/T cell ratio 1:10). An inhibitor of notch signaling (DAPT, 2.5 μM) was added to one half of the wells, vehicle (DMSO 1:10,000) to the other half. Supernatants were harvested after 96 h and analyzed for IL-2, IL-4, IL-5, IL-10 and IFN-γ by ELISA (OptEIA, Becton Dickinson, Heidelberg, Germany). Proliferation was assessed by ^3^H-thymidine incorporation.

### Statistics

To reveal statistically significant differences between treatment groups, Wilcoxon test for paired samples was employed. *P* values below 0.05 (*) were considered to indicate significance.

## Results

### Aqueous birch pollen extracts modulate the transcription of notch ligands in dendritic cells

**Figure 1:**
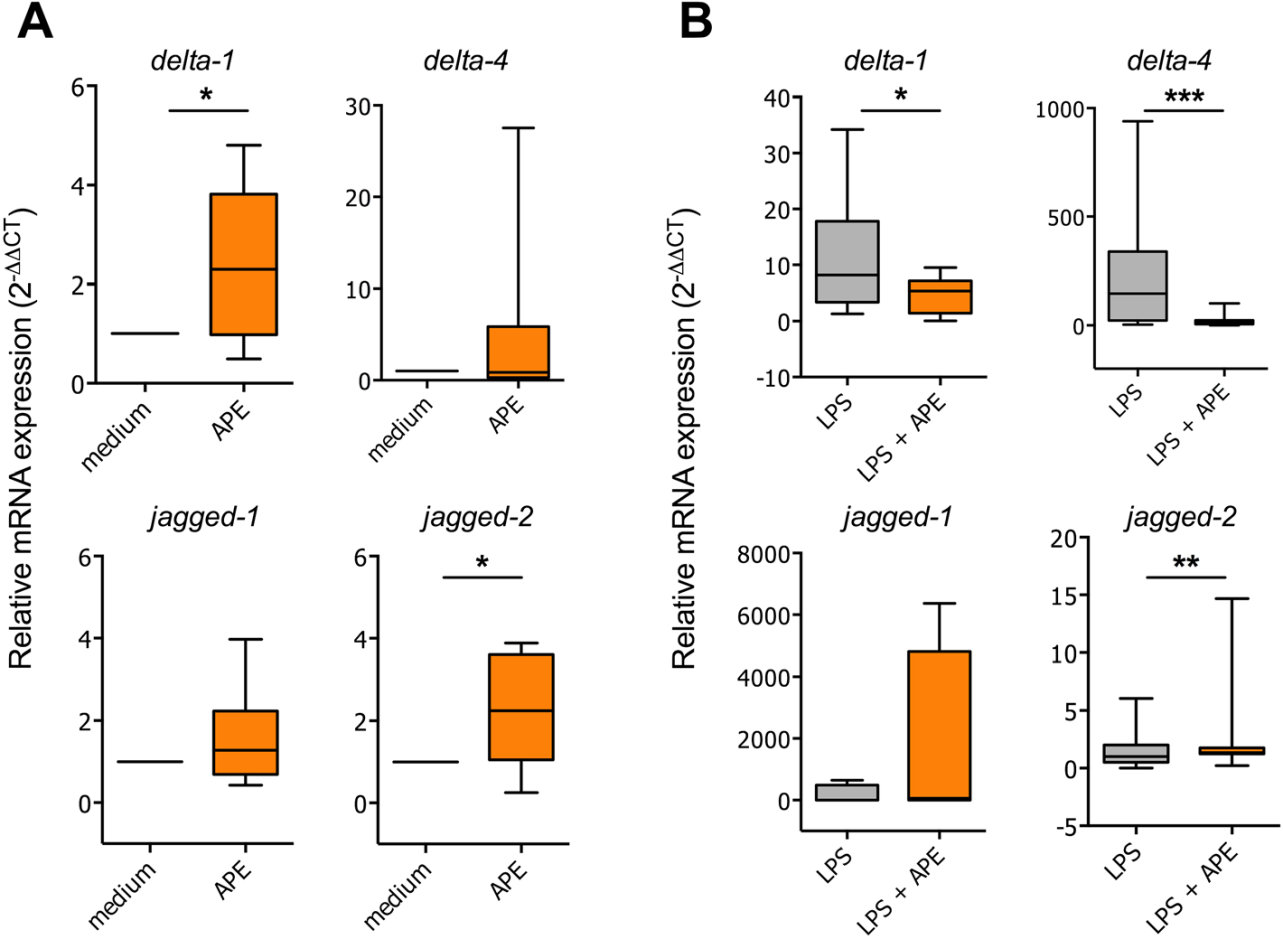
**Regulation of notch ligands** ***delta like*** **and** ***jagged*** **by aqueous birch pollen extracts.** Human monocyte-derived dendritic cells were incubated for 12 h in the presence of indicated stimulants (LPS: 100 ng/ml, APE: 10 mg/ml). **A**: Stimulation in the absence of LPS. **B**: Stimulation in the presence of LPS. Total RNA was extracted and cDNA subjected to qPCR using primers specific notch ligands *delta like-1*, −*4, jagged-1* and *jagged-2*. Relative mRNA expression is given as 2^-ΔΔCT^ with 18S RNA as house-keeping gene. Shown are the results of 4 (minimum) to 15 (maximum) independent experiments per condition. *: p < 0.05, two-tailed Wilcoxon signed rank test. APE: aqueous birch pollen extracts.

In immature dendritic cells, stimulation with aqueous birch pollen extracts (APE, 10 mg/ml) led to a slight but significant increase in the expression of notch ligands *delta like-1* and *jagged-2* (mean 2^-ΔΔCT^ ± SEM *delta like-1*: 2.4 ± 0.5; *jagged-2*: 2.2 ± 0.5). Expression levels of *delta like-4* and *jagged-1* in APE-stimulated cells did not differ significantly from unstimulated cells (mean 2^-ΔΔCT^ ± SEM *delta like-4*: 4.8 ± 3.0; *jagged-1*: 1.6 ± 0.4) (Figure 1A).

Maturation of dendritic cells for 12 h in the presence of LPS led to enhanced relative transcript levels of notch ligands *delta like-1* (mean 2^-ΔΔCT^ ± SEM: 11.1 ± 2.9), *delta like-4* (mean 2^-ΔΔCT^ ± SEM: 222.5 ± 73.3) and *jagged-1* (mean 2^-ΔΔCT^ ± SEM 163.6 ± 161.2). Relative mRNA levels of *jagged-2* were only moderately induced by LPS (mean 2^-ΔΔCT^ ± SEM: 1.5 ± 0.4) (Figure 1B). Stimulation of LPS-matured dendritic cells with APE resulted in significantly reduced transcript levels of notch ligands *delta like-1* and *delta like-4* (mean 2^-ΔΔCT^ ± SEM: *delta like-1*: 4.7 ± 0.9; *delta like-4*: 20.8 ± 7.2) as compared to cells stimulated with LPS alone. In contrast, *jagged-1* and *jagged-2* were induced by LPS plus APE as compared to LPS alone (mean 2^-ΔΔCT^ ± SEM: *jagged-1*: 1624.1 ± 1582.5; *jagged-2*: 2.7 ± 1.0) (Figure 1B). Up-regulation of *jagged-2* transcript levels in LPS/APE- compared to LPS-stimulated cells was confirmed in a separate experiment using 3 different reference genes, 18S, GAPDH and EF1α (mean 2^-ΔΔCT^ ± SEM: 18S-normalized: 3.8 ± 1.3; GAPDH-normalized: 2.3 ± 0.6; EF1α-normalized: 2.0 ± 0.4; see Additional file 2: Figure S2).

### Reduction of LPS-induced delta like-4 expression by aqeuous birch pollen extracts is dose-dependent and mediated by a low molecular-weight factor

**Figure 2:**
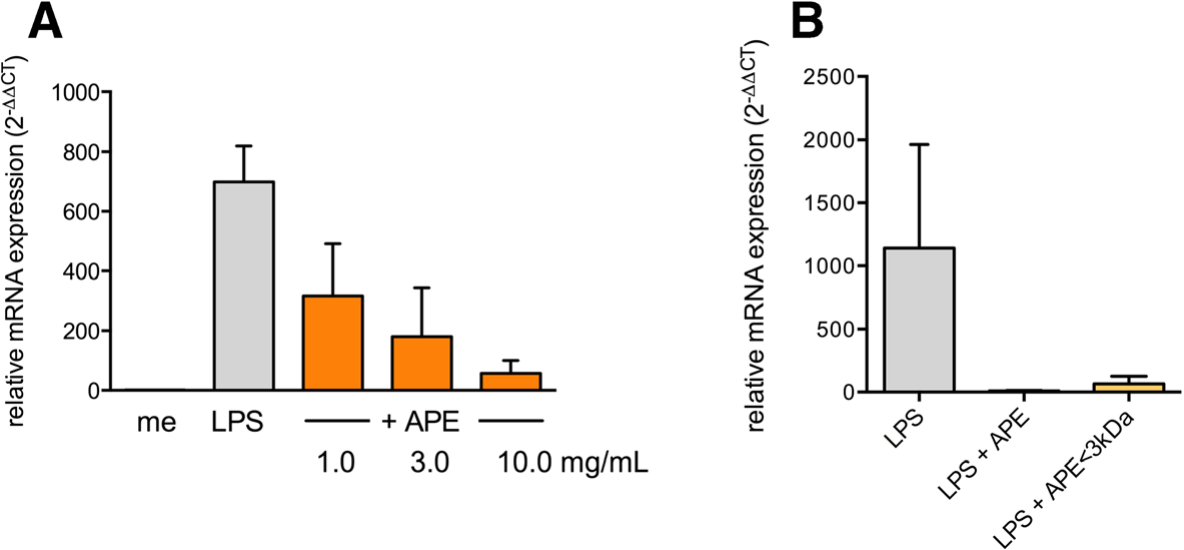
**Reduction of LPS-induced** ***delta like-4*** **mRNA expression by aqueous birch pollen extracts is dose-dependent and retained in a protein-free fraction. A:** Monocyte-derived dendritic cells were incubated for 12 h with medium, LPS (100 ng/mL) or LPS plus different concentrations of total aqueous birch pollen extract. Shown are mean + SD of 3 independent experiments. **B:** Monocyte-derived dendritic cells were treated for 12 h with medium, LPS (100 ng/mL), LPS plus APE (10 mg/mL) or LPS plus protein-free APE (APE < 3 kDa). Shown are mean values + SD of 3 independent experiments. Relative expression of *delta like-4* mRNA is expressed as 2^-ΔΔCT^. 18S RNA served as housekeeping gene. APE: aqueous pollen extract.

To investigate the nature of the Th1 inhibitory effect further, we focused on *delta like-4* expression as readout and exposed dendritic cells for 12 h to medium, LPS and LPS plus different concentrations of APE. As shown in Figure 2A, the inhibitory effect of APE on LPS-induced relative *delta like-4* mRNA expression was dose-dependent. We then analyzed whether a fraction of APE enriched for low molecular-weight factors is still capable of decreasing LPS-induced *delta like-4* expression. Aqueous birch pollen extracts were therefore separated by ultra-filtration using a 3 kDa cutoff filter device and applied either total extract (APE) or the filtrated fraction (APE < 3 kDa), enriched for molecules with a molecular mass of less than 3 kDa, to DCs in the presence of LPS. The result showed that the filtrate fraction of APE still contained the active component (Figure 2B).

### Regulation of Notch ligands and MyD88 is not mediated by E_1_-phytoprostanes

**Figure 3:**
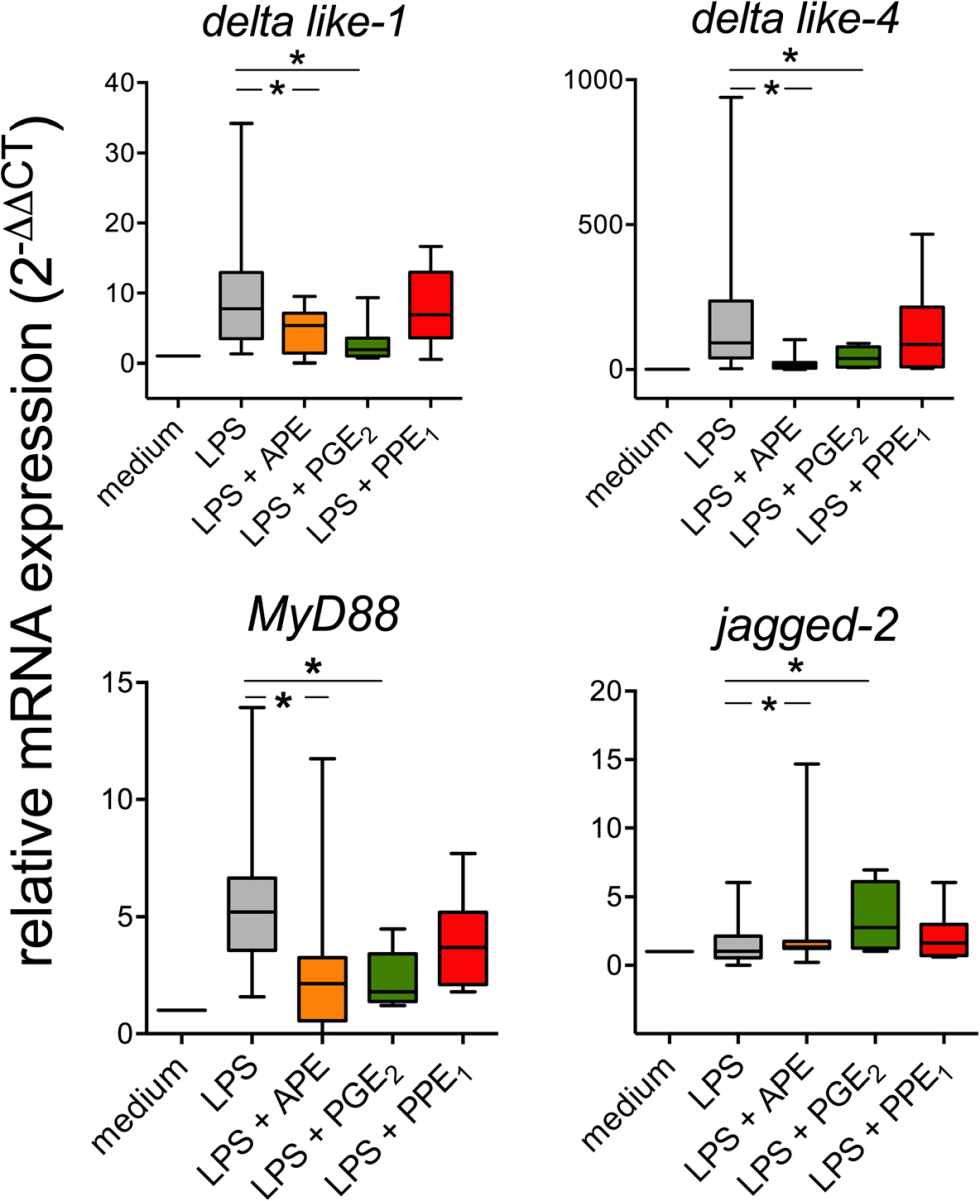
**Modulation of notch ligands and myD88 by aqueous birch pollen extracts is independent from pollen-derived of E**_**1**_ **-phytoprostanes.** Monocyte-derived dendritic cells were incubated for 12 h with medium, LPS (100 ng/mL), LPS plus APE (10 mg/mL), LPS plus PGE_2_ (10 μM) or LPS + PPE_1_ (1 μM). Relative mRNA expression of *delta like-1, delta like-4, jagged-2* and *myD88* were determined by real-time PCR and expressed as 2^-ΔΔCT^. 18S RNA served as house-keeping gene. Shown are the results of 5 (minimum) to 13 (maximum) independent experiments per condition. *: p < 0.05, two-tailed Wilcoxon signed rank test.

Since modulation of notch ligand *delta like-4* depended on a low molecular weight fraction of the pollen extract, we investigated whether pollen-derived E_1_-phytoprostanes could mediate the effect. PGE_2_, a lipid mediator known to inhibit *delta like* and to induce *jagged* notch ligands, was included as positive control in the analysis. Cells matured in the presence of LPS plus PGE_2_ showed a similar pattern of transcript regulation as cells matured in the presence of LPS plus APE: *myD88* and *delta like* notch ligands were reduced as compared to LPS alone (LPS/APE vs LPS; MyD88: 2.3 ± 0.6 vs 5.3 ± 1.3; *delta like-4*: 42.1 ± 14.4 vs 116.4 ± 40.1; *delta like-1*: 2.9 ± 1.0 vs 10.0 ± 2.6) while relative mRNA levels of *jagged-2* were induced (mean 2^-ΔΔCT^ ± SEM *jagged-2*: 3.3 ± 0.9 vs 2.2 ± 0.7) (Figure 3). E_1_-phytoprostanes (PPE_1_), pollen-associated lipid mediators with structural similarity to PGE_2_, did not have any significant effect on the LPS-induced expression of *myD88* or notch ligands (Figure 3). Mean relative expression levels (2^-ΔΔCT^ ± SEM) in DCs exposed to LPS plus PPE_1_ were 3.9 ± 0.9 (*myD88*), 137.1 ± 45.5 (*delta like-4*), 8.0 ± 1.9 (*delta like-1*) and 2.3 ± 0.7 (*jagged-2*).

### Extracts prepared from freshly collected birch pollen specimens modulate the LPS-induced expression of *myD88* and notch ligands

**Figure 4:**
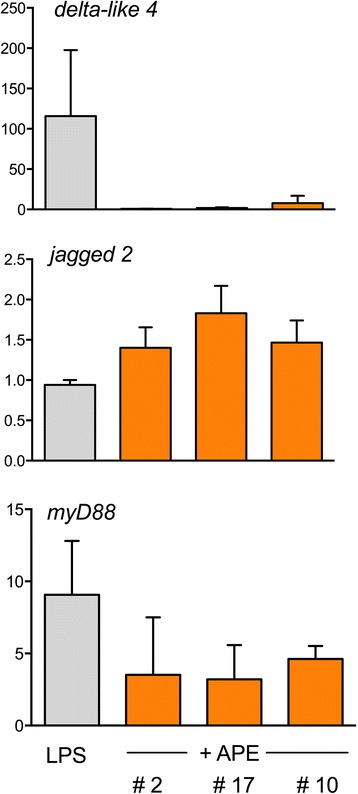
**Aqueous extracts from freshly collected birch pollen modulate the LPS-induced expression of notch ligands and MyD88.** Aqueous extracts were prepared from three different freshly collected birch pollen specimens (birch trees #2, #10, #17). Relative mRNA expression of *delta like-4, jagged-2* and MyD88 were determined by real-time PCR and expressed as 2^-ΔΔCT^. 18S RNA served as house-keeping gene. Shown are means + SEM of 3 independent experiments.

The aqueous extracts used in the study so far were prepared from birch pollen obtained from a commercial source. To exclude the possibility that the observed effects of APE on the expression of notch ligands *delta like-4* and *jagged-2* and on *myD88* are due to storage artefacts, we investigated whether they can be reproduced with extracts of freshly collected birch pollen. As seen in Figure 4, APE < 3 kDa prepared from pollen collected from three different birch trees during birch pollen season (April/May 2009) in Munich lowered the transcript rates of *myD88* and notch ligand *delta-like 4* while enhancing relative transcript levels of *jagged-2*.

### Aqueous birch pollen extracts decrease LPS-induced surface expression of Delta like-4 and maturation markers on dendritic cells

**Figure 5:**
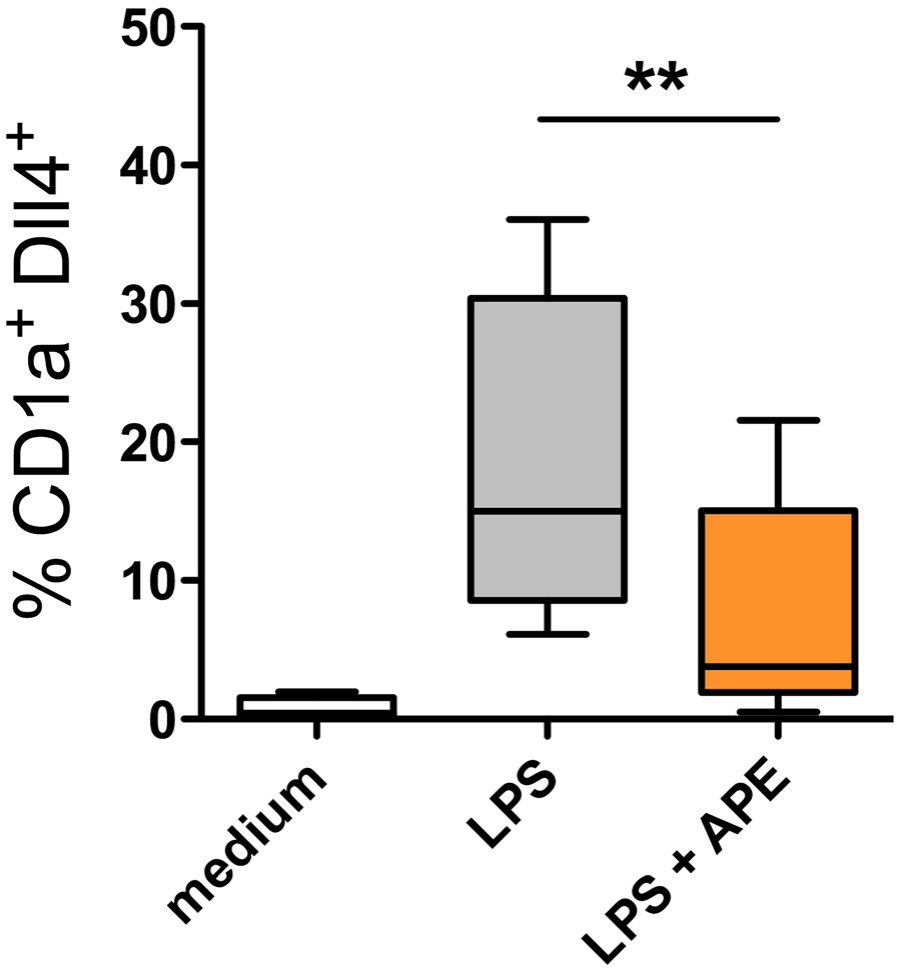
**Aqueous birch pollen extracts decrease LPS-induced Delta like-4 on protein level.** Dendritic cells were stimulated for 24 h with medium, LPS (100 ng/ml) or LPS + APE (10 mg/ml). Cells were stained with anti-CD1a and anti-Dll4 antibodies and analyzed by flow cytometry. Shown are percentages of double-positive cells from 9 independent experiments. **: p < 0.01, two-tailed Wilcoxon signed rank test. APE: aqueous pollen extract.

**Figure 6:**
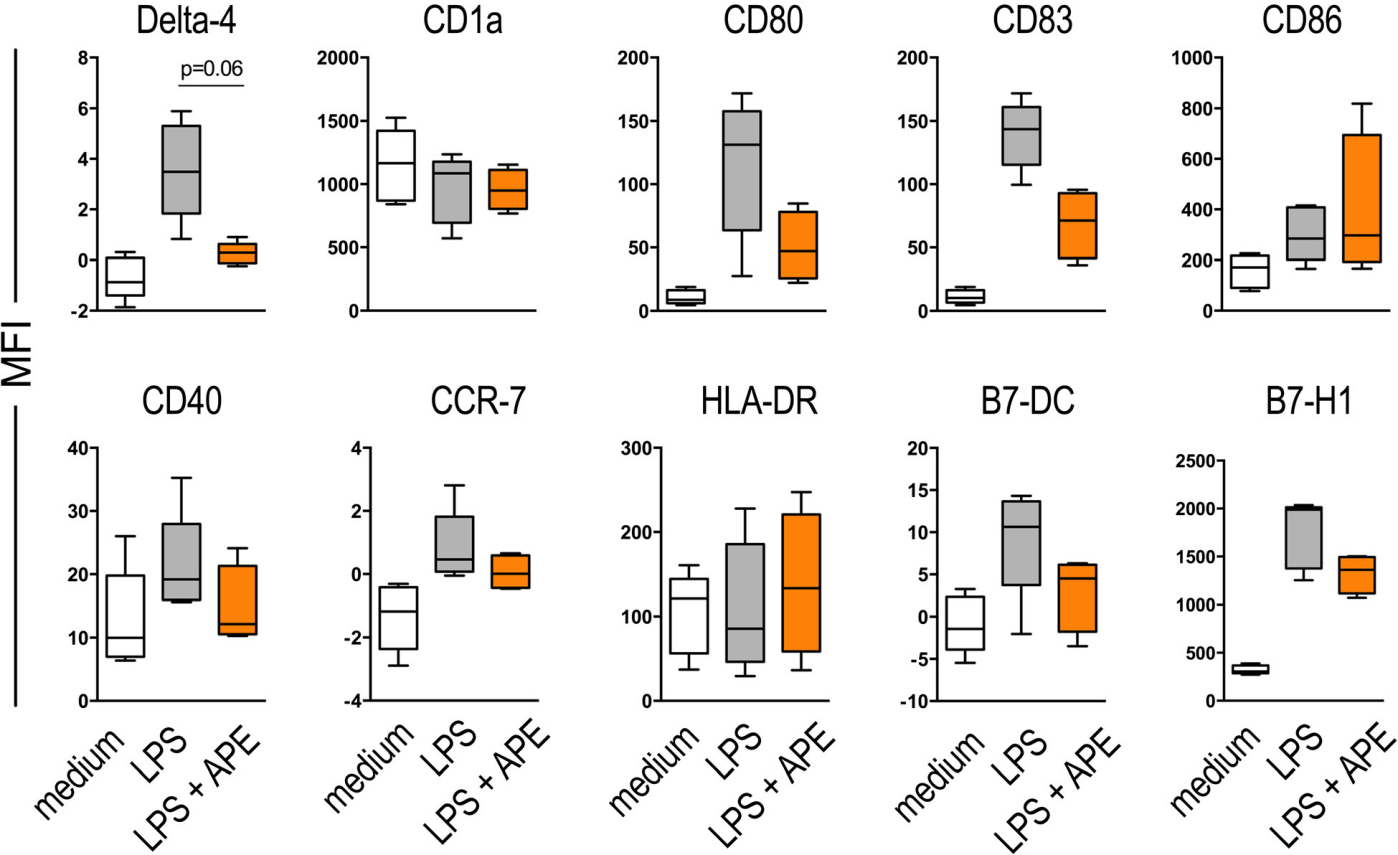
**Aqueous birch pollen extracts regulate the LPS-induced surface expression of Delta-4 and maturation markers on dendritic cells.** Dendritic cells were stimulated for 24 h with medium, LPS (100 ng/ml) or LPS + APE (10 mg/ml). Cells were stained with antibodies for indicated surface markers and subjected to multi-colour flow cytometry. Shown are mean fluorescence intensities (MFIs) + SEM of 5 independent experiments. MFIs of the respective isotype controls were subtracted. Results were not statistically significant (two-tailed Wilcoxon signed rank test).

To investigate whether the observed down-regulation of *delta like-4* mRNA in pollen-exposed DCs is mirrored by a down-regulation of Delta like-4 on protein level, we performed flow-cytometry of cells incubated for 24 h in the presence of medium, LPS or LPS plus ultra-filtered aqueous birch pollen extract (APE < 3 kDa). Surface staining of Delta like-4 protein revealed that Delta like-4 is not expressed on immature monocyte-derived DCs, while it is expressed on the surface of 5-30% of cells matured in the presence of LPS. In the presence of LPS plus APE < 3 kDa, this up-regulation is significantly reduced (Figure 5). The notch-ligands Jagged-1 and −2 were not detectable on protein level as determined by flow cytometry and Western blotting (data not shown). Mean fluorescence intensities (MFI) specific for Delta like-4 and maturation markers CD80, CD83, CD40, CCR-7, B7-DC and B7-H1 (Figure 6) were lower in DCs matured in the presence of LPS plus APE as compared to LPS DCs, however the differences between LPS and LPS/APE did not reach statistic significance. LPS induced expression of HLA-DR remained unchanged by APE and CD1a expression was not regulated by LPS or LPS plus APE.

### Low molecular-weight factors from pollen decrease LPS-induced MyD88 expression in the cytoplasm of dendritic cells

**Figure 7:**
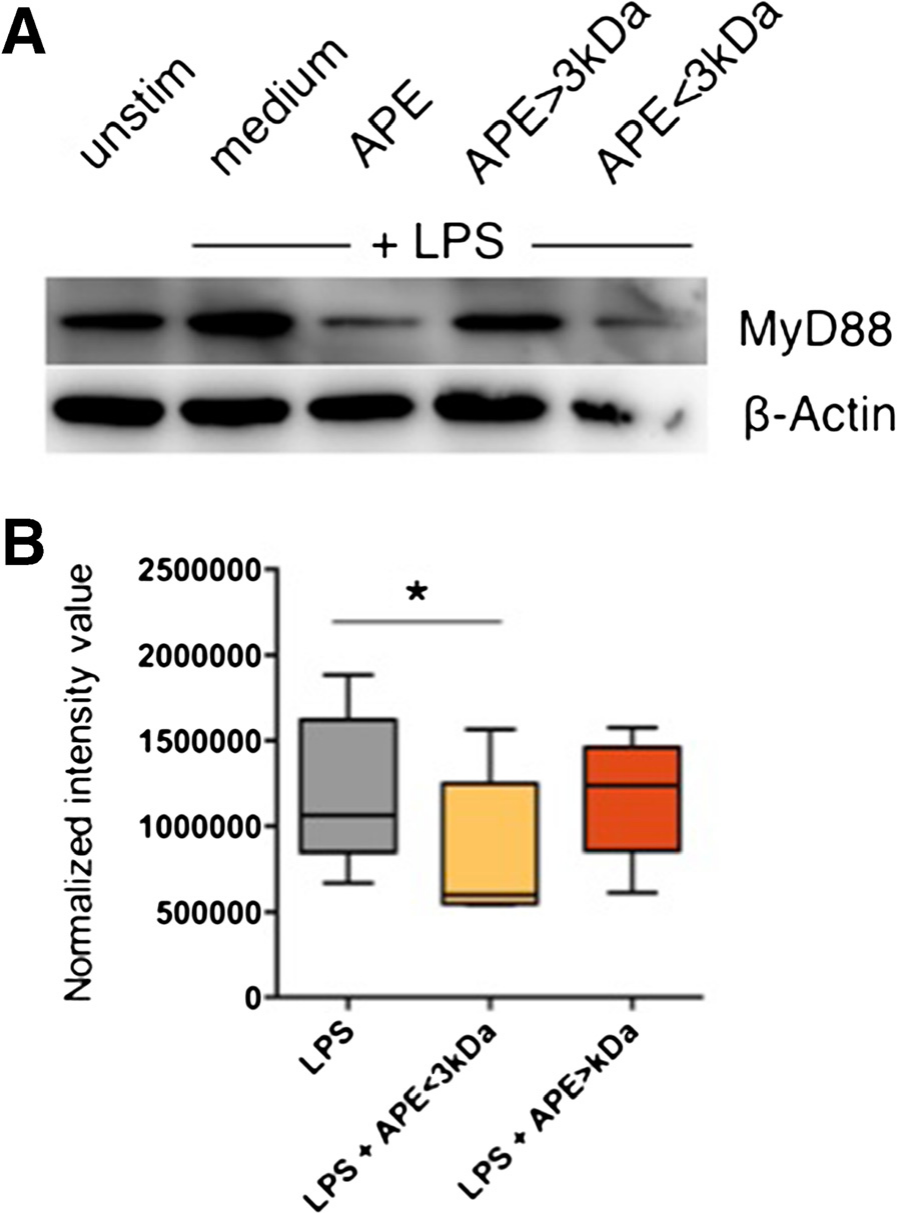
**Aqueous birch pollen extracts reduce cytoplasmic expression of LPS-induced MyD88.** Dendritic cells were stimulated for 24 h with medium, LPS (100 ng/ml), LPS + APE (10 mg/ml), LPS + APE < kDa (10 mg/ml) or LPS + APE > 3 kDa (10 mg/ml). Cytoplasmic lysates were subjected to SDS PAGE and Western blotting, and MyD88 was detected in the lysates by an anti-MyD88 antibody. **A:** Depicted is the result of one representative experiment. **B:** Quantitation of MyD88 relative to β-actin bands. Shown are results of 6 independent experiments. APE: aqueous pollen extract. *: p < 0.05, two-tailed Wilcoxon signed rank test.

In cytoplasmic extracts of DCs matured with LPS, an up-regulation of MyD88 protein was detected by Western blotting (Figure 7, A, lanes 1–2). This was reduced in cells exposed to APE as well as to the low molecular weight fraction, APE < 3 kDa (Figure 7, A, lanes 3 and 5), but not to the high molecular weight fraction, APE > 3 kDa (Figure 7, A, lane 4). Figure 7, B shows quantitation of MyD88 protein normalized to β-actin.

### Inhibition of notch signaling results in increased Th2 polarization in allogeneic DC/T cell co-cultures stimulated by aqueous pollen extracts

**Figure 8:**
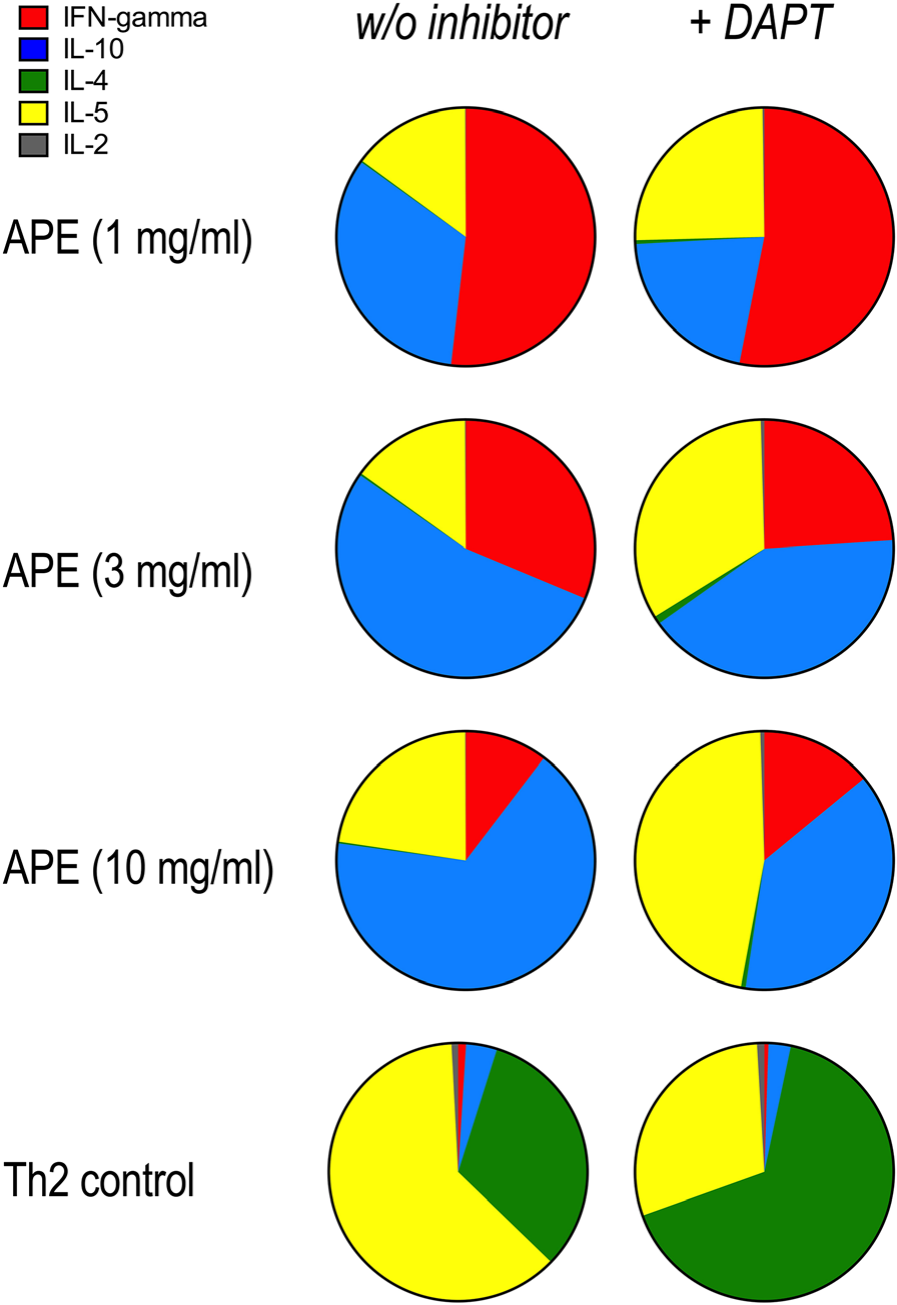
**Notch signaling modulates cytokine production in response to pollen in allogeneic T cell stimulation assays.** Dendritic cells were stimulated with different concentrations of aqueous birch pollen extracts (APE) or a Th2 differentiation cocktail and co-cultured for 4 days with allogeneic naïve CD4^+^ T cells in the absence or presence of the γ-secretase inhibitor DAPT. Cytokines IL-2, IL-4, IL-5, IL-10 and IFN-γ were measured in supernatants by ELISA. Shown are mean cytokine levels of 4 (Th2 controls) or 5 (APEs) independent experiments.

To analyze the role of notch signaling in pollen-stimulated dendritic cells for the polarization of T helper cell responses, monocyte-derived DCs were stimulated with aqueous birch pollen extracts (APE) at different concentrations and co-cultured with allogeneic, naïve CD4^+^ T cells in the absence or presence of the γ-secretase inhibitor DAPT. Mean cytokine levels (pg/ml ± SD) in the supernatants of co-cultures were: “Th2 control”: 233.5 ± 196.7 (IL-4), 457.0 ± 264.6 (IL-5), 175.8 ± 86.3 (IL-10), 65.6 ± 27.8 (IFN-γ); “APE, 10 mg/ml”: 2.0 ± 2.0 (IL-4), 124.5 ± 89.4 (IL-5), 357.6 ± 158.4 (IL-10), 52.8 ± 21.7 (IFN-γ). Functionality of DAPT was demonstrated by selective blocking of IFN-γ in Th1 control cells (Additional file 3: Figure S3). To assess cytokine profiles in the DC/T cell co-cultures, cytokine release was normalized to proliferation. As illustrated in Figure 8, mixed allogeneic co-cultures of CD4^+^ T cells and DCs pre-stimulated with rising APE concentrations (1 - 10 mg/ml) produce a mixed cytokine profile consisting of IFN-γ, IL-10 and IL-5. Th2 controls produce low amounts of IFN-γ, some IL-2 and IL-10 and high amounts of IL-5 and IL-4. In the absence of DAPT, APE induces a dose-dependent decrease of IFN-γ and a dose-dependent increase in IL-10 whereas IL-5 levels do not change. In the presence of DAPT, APE still induces a dose-dependent decrease in IFN-γ. However, IL-10 levels are lower than in the absence of DAPT, whereas IL-5 levels are increased. Under Th2 driving conditions (“Th2 control”), DAPT lowers IL-5 and enhances IL-4 production while other cytokines remain unaffected. Additional file 4: Figure S4 shows mean cytokine levels, normalized to proliferation.

## Discussion

Previous studies of the immuno-modulatory potential of pollen focused on the modulation of dendritic cell cytokine release, foremost on modulation of the key Th1 instructing cytokine IL-12. Besides inhibiting Th1 differentiation, dendritic cells exposed to aqueous pollen extracts (APE) were also shown to promote the differentiation of Th2 cells [11,13]. A more recent study revealed adenosine within a low molecular weight fraction of aqueous birch pollen extracts, conferring tolerogenic properties on dendritic cells derived from non-atopic individuals [21].

The current study shows for the first time that pollen-derived water-soluble mediators within aqueous pollen extracts (APE) modulate the expression of membrane-bound ligands involved in polarizing T cell responses. In addition it demonstrates that APE reduces the LPS-induced expression of the TLR signaling molecule MyD88. Modulation of notch ligands and MyD88 occurs independently of the previously identified E_1_-phyoprostanes but are mediated by a fraction of APE enriched for substances with a molecular weight below 3 kDa (APE < 3 kDa), suggesting the involvement of a non-protein compound. Finally, we demonstrate the involvement of notch signaling in pollen-exposed dendritic cells in the initiation of T helper cell responses.

Although IL-4 is well established as Th2 promoting cytokine, a defined dendritic cell-derived factor inducing Th2 differentiation has not been identified. Presently it is thought that microenvironmental factors like PGE_2_, TSLP and IL-4 derived from cells other than DCs, e.g. innate lymphoid cells, supply the signals for the development of Th2 cells. An alternative assumption is that lack of DC-derived IL-12p70 during DC/T cell interaction leads to the development Th2 cells by a default differentiation pathway (reviewed in [18]). Not until 2004, Amsen et al. showed that differential expression of notch ligands *delta* and *jagged* on DCs can directly instruct T helper cells to become Th1 or Th2 cells, respectively [15]. The importance of Jagged ligands on DCs in eliciting IL4 in naïve T helper cells was confirmed in several studies, one of them demonstrating the contribution of jagged 1/notch interaction in an *in vivo* allergic lung inflammation model [22,23]. Later on, another receptor/ligand pair, TIM4 on the DC and TIM1 on the T cell, was shown to maintain Th2-skewing in allergic rhinitis patients [24], further emphasizing the role of cell-to-cell contact dependent mechanisms in the regulation of T helper cell differentiation.

We report here that APE moderately reduced the LPS-mediated up-regulation of co-stimulatory molecules (CD40, B7 family members CD80, B7-H1, B7-DC) on the surface of monocyte-derived DCs. Birch pollen extracts were previously shown to block LPS induced maturation of 6-sulfo-LacNAc^+^ DCs, altering their T cell differentiating potential [25]. The role of B7 familiy proteins in skewing T helper cell differentiation remains ambigous [26-28]. However, up-regulation of Delta-like 4 on the surface of maturing dendritic cells was significantly impaired by APE. This suggests that the Th1-differentiating potential might be reduced in pollen-exposed dendritic cells.

The expression of notch ligands on the dendritic cell depends on MyD88 [15]. Consecutively, synergistic TLR engagement was shown to induce a sustained Th1-promoting DC phenotype distinguished by enhanced proinflammatory cytokine release and an elevated ratio of *delta*/*jagged* mRNA expression [29]. To date, there is abundant evidence for a role of notch signaling in shaping the outcome of T helper cell responses [15-18,29-34]. Particularly the ratio of *delta/jagged* expression seems to be associated with the potential of dendritic cells to induce Th1 or Th2 responses [29].

Aqueous birch pollen extracts do not only mediate inhibition of LPS-induced *delta like-1* and −*4* but also induce the Th2-instructing ligands *jagged-2* and, to a lesser degree, *jagged-1*. This finding supports the notion that, along with the allergens, pollen deliver signals that actively drive the adaptive immune response towards Th2. Modulation of *jagged* mRNA expression after combined TLR engagement has been shown to follow a slower kinetic than modulation of *delta like-4* (maximum after 12 h), with maximal regulation after 24 h [29]. However, we were not able to detect Jagged proteins in lysates or on the surface of cells stimulated for 24 h (data not shown). This indicates that Jagged proteins are not expressed at high enough levels in monocyte-derived dendritic cells under the conditions tested. Interestingly, timothy grass pollen extracts were recently shown to regulate trancription of notch signaling pathway genes in airway epithelial cells, however, in contrast to our results, this study found *jagged* transcripts to be down-regulated [35]. This discrepancy might reflect differences in the composition of extracts of different pollen species.

E_1_-phyoprostanes (PPE_1_), a previously identified class of PALMs known to inhibit the dendritic cell’s IL-12 production, were analyzed as candidate substance for the modulation notch ligand expression. We previously demonstrated that PPE_1_ inhbit IL12p70 by interfering with NFκB signaling in DCs [10]. NFκB is a downstream molecule in TLR signaling and might be affected either directly or indirectly, e. g. via suppression of MyD88. However, PPE_1_ did not influence expression of notch ligands. This result suggests that E_1_-phytoprostanes inhibit NF-kB signaling downstream of MyD88, which is in agreement with our previous finding that inhibition of IL12p70 by PPE_1_ depends on peroxisome proliferator-activated receptor(PPAR)-γ [10]. There are several independent hints that modulation of DC cytokine and chemokine expression by aqueous pollen extracts can occur independently of pollen-derived PPE_1_, e. g. by induction of cyclic AMP signaling [10,12].

Notably, aqueous birch pollen extracts not only inhibited expression of *delta like* notch ligands but also of the TLR adapter MyD88. MyD88 transmits multiple proinflammatory and Th1-instructing signals following TLR engagement. In dendritic cells, both secretion of the Th1-promoting cytokine IL12p70 and surface expression of the Th1-instructing notch ligand Delta-like 4 depend on MyD88. When functional MyD88 is missing, DCs display an altered response to “classical” Th1-promoting stimuli: while general maturation programs seem not to be impaired, DCs fail to prime Th1 responses after exposure to LPS or respiratory syncytial virus (RSV). Instead, Th2 responses are mounted to the same stimuli [31,36]. The finding that pollen-derived low molecular weight factors inhibit expression of MyD88 in dendritic cells adds further evidence to the concept of Th2-driven responses to pollen: On one hand, allergens are delivered to the mucosal immune system in conjunction with PAMPs and danger signals, such as bacterial and fungal cell-wall components, plant secondary metabolites and pollen-associated lipid mediators (PALMs). This might be mirrored by the finding that during birch pollen season, proinflammatory chemokine genes are gradually up-regulated in nasal epithelium of non-sensitized individuals [37]. On the other hand, pollen release substances which down-regulate a key signal transducing molecule in innate danger sensing, MyD88. Under natural exposure conditions, i.e. in the presence of pollen-derived danger signals, this suppression of proinflammatory and Th1-promoting cellular programs might promote the induction of Th2-biased immune responses. Several MyD88 signaling TLR ligands, such as TLR3, TLR7 and TLR9, are also expressed in nasal epithelium [38]. It might therefore be of future interest to study the impact of pollen-derived low molecular weight substances on the nasal epithelial immune response to PAMPs.

The results of our allogeneic T cell stimulation assays demonstrate that notch signaling in DCs and T cells contributes to shaping the outcome of T helper cell response to pollen. Blocking of notch signaling was previously shown to inhibit Th1 differentiation *in vitro* and in animal models [16,39,40]. DAPT works by inhibiting gamma secretase and therefore blocks translocation of notch intracellular domain to the nucleus. Since notch was shown to bind directly to and activate the tbx1 promoter [16], blocking of notch is thought to lead to reduced expression of t-bet, which results in reduced IFN-γ production. In our model, DAPT blocked IFN-γ production of Th1 cell lines by 50%. In co-cultures of T cells and APE-stimulated dendritic cells DAPT shifted the overall cytokine profile towards a more Th2 skewed response, which suggests a contribution of notch signaling in dendritic cell driven T cell responses to pollen.

## Conclusion

Taken together, the present study shows that non-protein substance(s) released from pollen inhibit early events in TLR signaling, co-stimulatory molecules and T helper cell instructing notch ligands on the cell surface, contributing to the Th2 biasing potential of pollen.

## Additional files

Additional file 1: Figure S1. Levels of Bet v 1 and endotoxin in aqueous birch pollen extracts and a protein-free fraction thereof. A: Bet v 1 levels in aqueous extracts prepared from commercial birch pollen (Allergon) were lower than in aqueous extracts prepared from freshly collected birch pollen. In ultra-filtered fractions of aqueous birch pollen extracts (APE < 3 kDa), no Bet v 1 was detected. B: Endotoxin levels as detemined by LAL-assay were 0.27 EU/ml in aqueous extracts prepared from Allergon pollen whereas they were significantly higher in extracts prepared from freshly collected pollen. In APE < 3 kDa endotoxin levels were below the detection limit. Additional file 2: Figure S2. Up-regulation of jagged-2 mRNA expression in relation to different house-keeping genes. Human monocyte-derived dendritic cells were incubated for 12 h in the presence of LPS or a combination of LPS and APE (LPS: 100 ng/ml, APE: 10 mg/ml). Total RNA was extracted and cDNA subjected to qPCR using primers specific notch ligand *jagged-2*. Relative mRNA expression is given as 2^-ΔΔCT^ with GAPDH (left panel), EF1α (middle panel) and 18S RNA (right panel) as house-keeping genes. Shown are the results of 5 independent experiments. *: p < 0.05, two-tailed Wilcoxon signed rank test. APE: aqueous birch pollen extracts. Additional file 3: Figure S3. The γ-secretase inhibitor DAPT selectively blocks IFN-γ production in a Th1 cell line. Immature dendritic cells were stimulated for 24 h with LPS (100 ng/ml) plus human recombinant IFN-γ (100U/ml). DCs were then co-cultured with allogeneic, naïve CD4^+^ T cells under addition of human recombinant IL-12p70 (250 ng/ml) and a blocking anti-IL-4 antibody (10 μg/ml) in the absence or presence of DAPT (2.5 μM). At day 4, supernatants were analyzed for the cytokines IL-4, IL-5, IL-10 and IFN-γ by ELISA. Shown are mean cytokine levels + SEM of 4 biological replicates. Additional file 4: Figure S4. Cytokine production in DC/T cell co-cultures in absence or presence of γ-secretase inhibitor. Dendritic cells were stimulated with different concentrations of aqueous birch pollen extract (APE, 1-10 mg/ml) or a Th2 differentiation cocktail and co-cultured for 4 days with allogeneic naïve CD4^+^ T cells in the absence or presence of DAPT (2.5 μM). Cytokines IL-4, IL-5, IFN-γ, IL-10 and IL-2 were measured in supernatants by ELISA. Proliferation was measured by ^3^H-thymidine incorporation. Shown are mean cytokine levels + SEM of 4 (Th2 controls) or 5 (APEs) independent experiments. *: p < 0.05, two-tailed Wilcoxon signed rank test.

## Abbreviations

DC: Dendritic cell
Dll1: Delta like-1
Dll4: Delta like-4
DAPT: *N*-[(3,5-Difluorophenyl)acetyl]-L-al anyl-2-phenyl]glycine-1,1-dimethylethyl ester (γ-secretase inhibitor)
APE: Aqueous pollen extract(s)
PPE_1_: E_1_-phytoprostanes
PGE_2_: Prostaglandin E_2_
PALM: Pollen-associated lipid mediator
TSLP: Thymic Stromal-derived Lymphopoietin
TLR: Toll-like receptor
MyD88: Myeloid differenciation primary response protein 88
