# Supplementary figures and images for: Non-allergenic factors from pollen modulate T helper cell instructing notch ligands on dendritic cells
Source: World Allergy Organ J. 2015 Jan 20;8(1):2. doi: 10.1186/s40413-014-0054-8 (PMC4300172; doi:10.1186/s40413-014-0054-8)

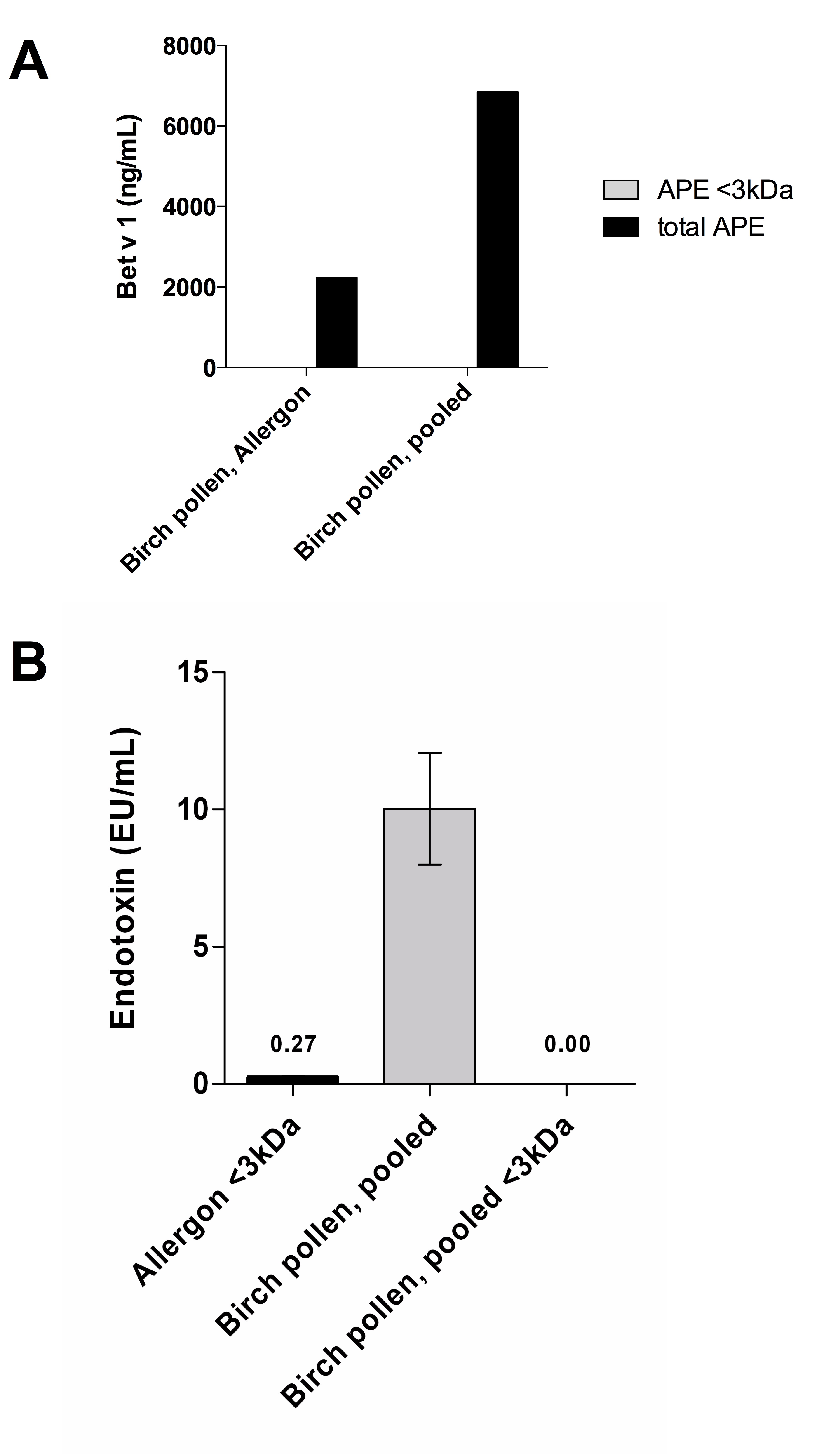

Supplement: Additional file 1: Figure S1. — Levels of Bet v 1 and endotoxin in aqueous birch pollen extracts and a protein-free fraction thereof. A: Bet v 1 levels in aqueous extracts prepared from commercial birch pollen (Allergon) were lower than in aqueous extracts prepared from freshly collected birch pollen. In ultra-filtered fractions of aqueous birch pollen extracts (APE < 3 kDa), no Bet v 1 was detected. B: Endotoxin levels as detemined by LAL-assay were 0.27 EU/ml in aqueous extracts prepared from Allergon pollen whereas they were significantly higher in extracts prepared from freshly collected pollen. In APE < 3 kDa endotoxin levels were below the detection limit. [file 40413_2014_54_MOESM1_ESM.jpeg]

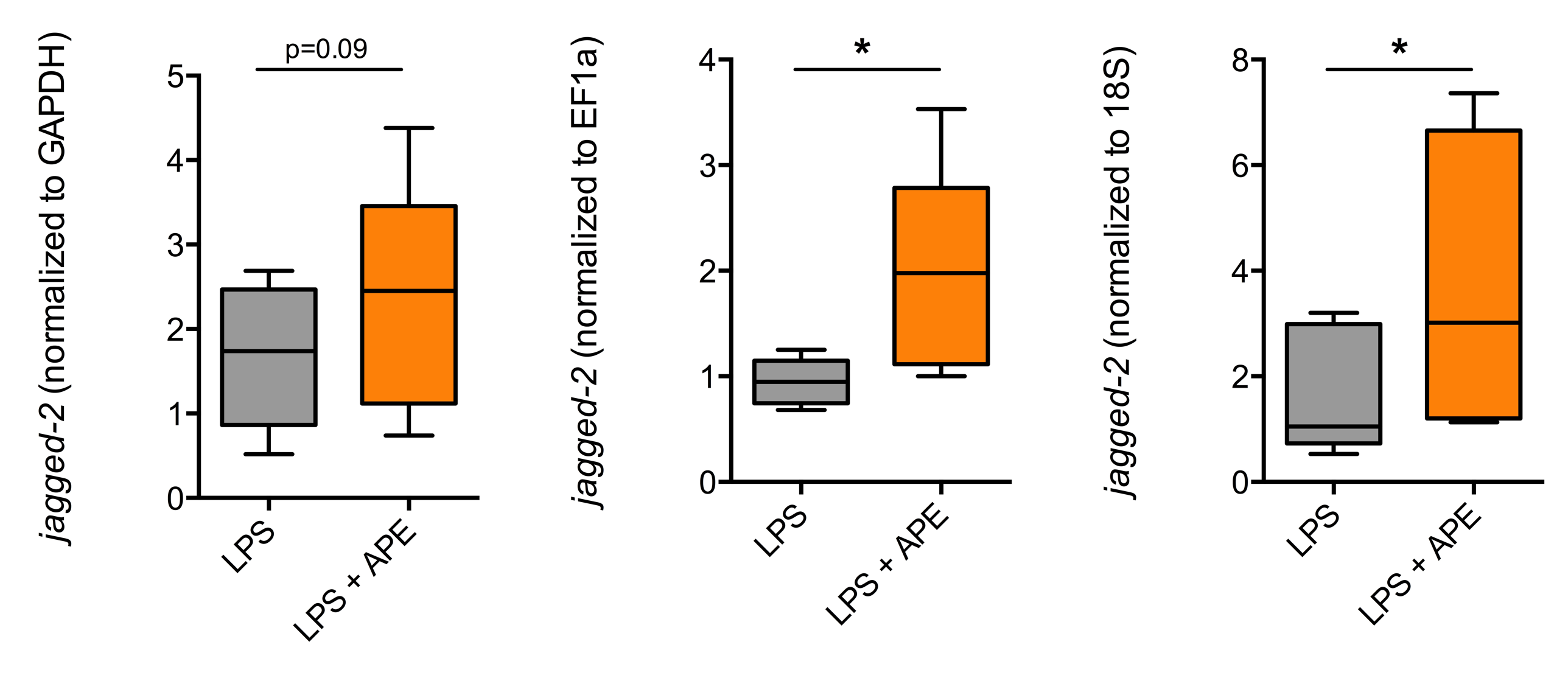

Supplement: Additional file 2: Figure S2. — Up-regulation of jagged-2 mRNA expression in relation to different house-keeping genes. Human monocyte-derived dendritic cells were incubated for 12 h in the presence of LPS or a combination of LPS and APE (LPS: 100 ng/ml, APE: 10 mg/ml). Total RNA was extracted and cDNA subjected to qPCR using primers specific notch ligand jagged-2. Relative mRNA expression is given as 2-ΔΔCT with GAPDH (left panel), EF1α (middle panel) and 18S RNA (right panel) as house-keeping genes. Shown are the results of 5 independent experiments. *: p < 0.05, two-tailed Wilcoxon signed rank test. APE: aqueous birch pollen extracts. [file 40413_2014_54_MOESM2_ESM.jpeg]

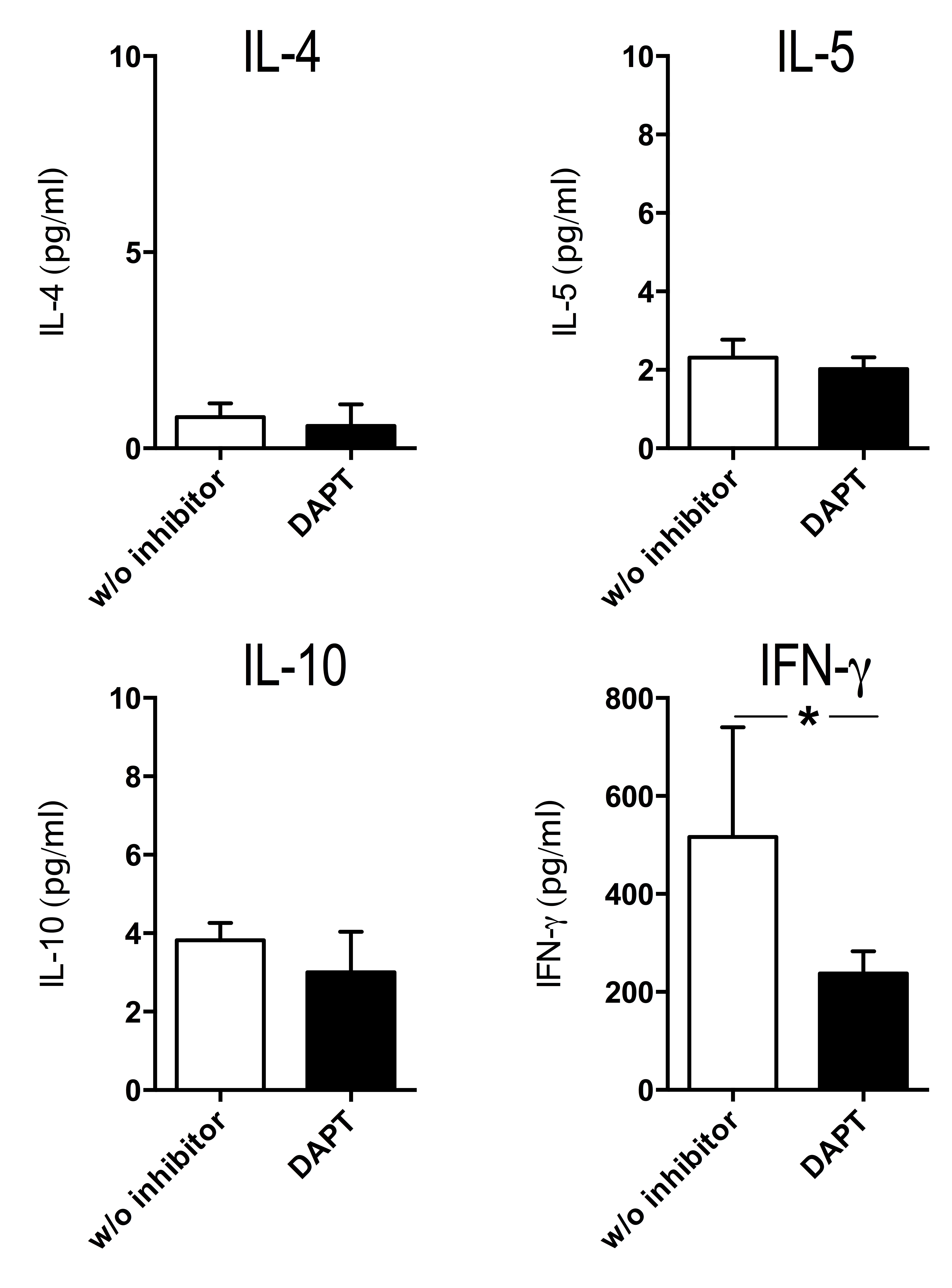

Supplement: Additional file 3: Figure S3. — The γ-secretase inhibitor DAPT selectively blocks IFN-γ production in a Th1 cell line. Immature dendritic cells were stimulated for 24 h with LPS (100 ng/ml) plus human recombinant IFN-γ (100U/ml). DCs were then co-cultured with allogeneic, naïve CD4+ T cells under addition of human recombinant IL-12p70 (250 ng/ml) and a blocking anti-IL-4 antibody (10 μg/ml) in the absence or presence of DAPT (2.5 μM). At day 4, supernatants were analyzed for the cytokines IL-4, IL-5, IL-10 and IFN-γ by ELISA. Shown are mean cytokine levels + SEM of 4 biological replicates. [file 40413_2014_54_MOESM3_ESM.jpeg]

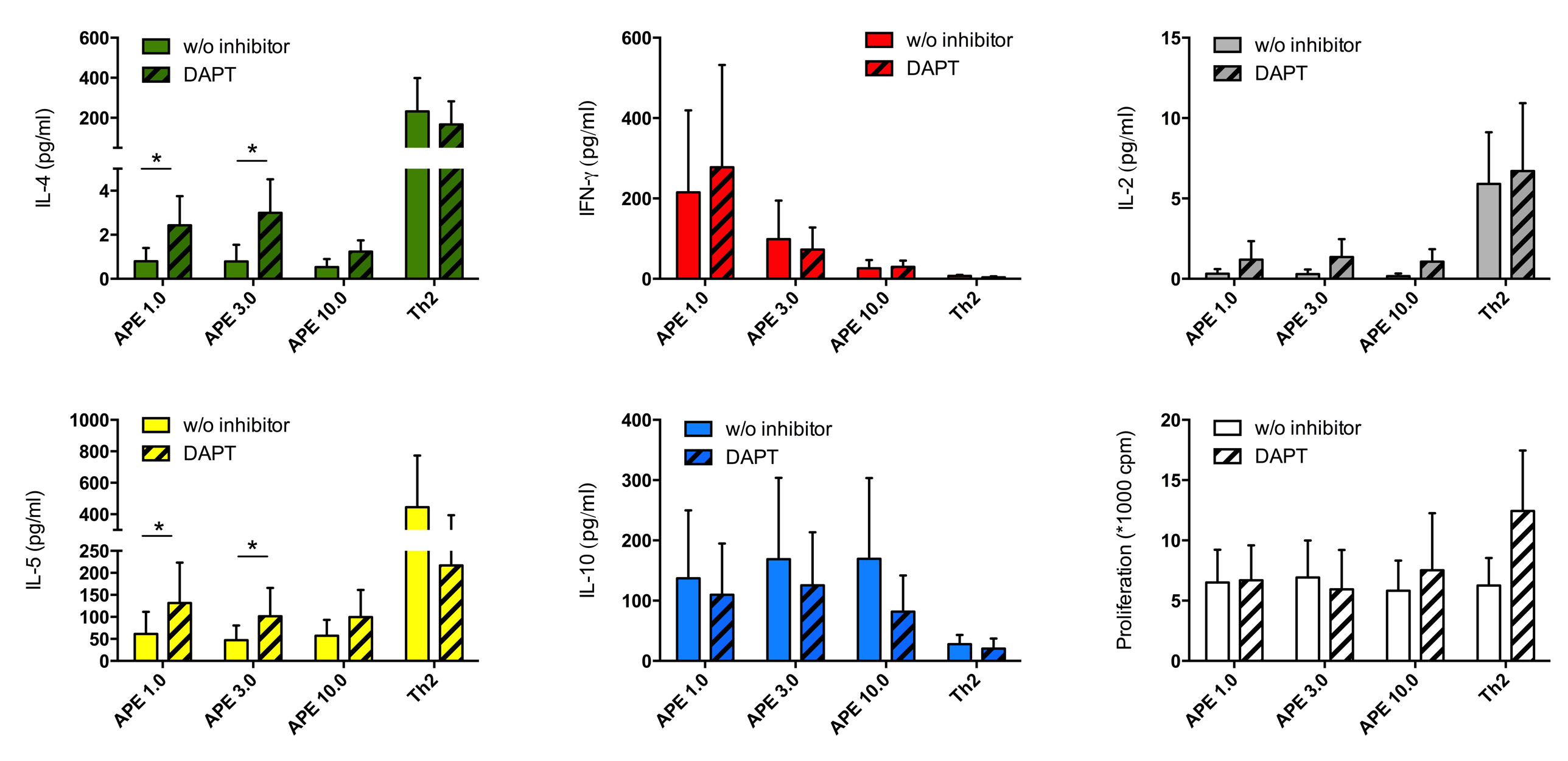

Supplement: Additional file 4: Figure S4. — Cytokine production in DC/T cell co-cultures in absence or presence of γ-secretase inhibitor. Dendritic cells were stimulated with different concentrations of aqueous birch pollen extract (APE, 1-10 mg/ml) or a Th2 differentiation cocktail and co-cultured for 4 days with allogeneic naïve CD4+ T cells in the absence or presence of DAPT (2.5 μM). Cytokines IL-4, IL-5, IFN-γ, IL-10 and IL-2 were measured in supernatants by ELISA. Proliferation was measured by 3H-thymidine incorporation. Shown are mean cytokine levels + SEM of 4 (Th2 controls) or 5 (APEs) independent experiments. *: p < 0.05, two-tailed Wilcoxon signed rank test. [file 40413_2014_54_MOESM4_ESM.jpeg]
